# Molecular Weight-Dependent Activity of Aminated Poly(α)glutamates as siRNA Nanocarriers

**DOI:** 10.3390/polym10050548

**Published:** 2018-05-20

**Authors:** Adva Krivitsky, Vadim Krivitsky, Dina Polyak, Anna Scomparin, Shay Eliyahu, Hadas Gibori, Eilam Yeini, Evgeni Pisarevsky, Rachel Blau, Ronit Satchi-Fainaro

**Affiliations:** 1 Department of Physiology and Pharmacology, Sackler Faculty of Medicine, Room 607, Tel Aviv University, Tel Aviv 69978, Israel; advashy@gmail.com (A.K.); dina.polyak@gmail.com (D.P.); anna.scomparin@gmail.com (A.S.); s_eliahu@hotmail.com (S.E.); hadas.gibori@gmail.com (H.G.); eilamyeini@mail.tau.ac.il (E.Y.); Jalchemic@gmail.com (E.P.); rachelniss@gmail.com (R.B.); 2 School of Chemistry, the Raymond and Beverly Sackler Faculty of Exact Sciences, Tel-Aviv University, Tel Aviv 69978, Israel; vadimkri@gmail.com; 3 Department of Neurosurgery, Stanford University School of Medicine, Stanford, CA 94305, USA

**Keywords:** polyplexes, siRNA delivery, physico-chemical characterization, cationic polymer, anticancer therapy, poly(α)glutamate

## Abstract

RNA interference (RNAi) can contribute immensely to the area of personalized medicine by its ability to target any gene of interest. Nevertheless, its clinical use is limited by lack of efficient delivery systems. Polymer therapeutics can address many of the challenges encountered by the systemic delivery of RNAi, but suffer from inherent drawbacks such as polydispersity and batch to batch heterogeneity. These characteristics may have far-reaching consequences when dealing with therapeutic applications, as both the activity and the toxicity may be dependent on the length of the polymer chain. To investigate the consequences of polymers’ heterogeneity, we have synthesized two batches of aminated poly(α)glutamate polymers (PGAamine), differing in their degree of polymerization, but not in the monomer units or their conjugation. Isothermal titration calorimetry study was conducted to define the binding affinity of these polymers with siRNA. Molecular dynamics simulation revealed that Short PGAamine:siRNA polyplexes exposed a higher amount of amine moieties to the surroundings compared to Long PGAamine. This resulted in a higher zeta potential, leading to faster degradation and diminished gene silencing. Altogether, our study highlights the importance of an adequate physico-chemical characterization to elucidate the structure–function-activity relationship, for further development of tailor-designed RNAi delivery vehicles.

## 1. Introduction

RNA interference (RNAi) has an enormous therapeutic potential due to its ability to silence disease-causing genes [1,2]. mRNA cleavage resulting from the introduction of matching small interference RNA (siRNA) sequences can be especially beneficial as a targeted therapy for cancer, but has failed to cross the barrier from bench to bedside due to lack of efficient delivery systems. Without a delivery vehicle, siRNAs are poorly internalized to cells, have short half-life in biological fluids, rapidly degrade in the bloodstream, or otherwise are excreted when systemically administered. In addition, siRNAs are highly immunogenic, suffer from off-target activity, and their activity may be altered by inappropriate cellular compartmentalization [3,4,5]. The integration of siRNA in nanoparticles can address many of the challenges mentioned, and furthermore, “passively” (extravasation-dependent) target the tumor site via the enhanced permeability and retention (EPR) effect, characterized by the leakiness of blood vessels and poor lymphatic drainage at the tumor site [6,7]. Various RNAi delivery approaches, such as liposomal complexes (lipoplexes), encapsulation in nanoparticles or polymeric complexes (polyplexes) were developed, from which none met the strict criteria for FDA approval, mostly due to low efficacy and high toxicity [8,9,10,11,12].

Polymer therapeutics is the term coined to describe pharmacologically-active entities composed of various chemical functionalities bound to a nano-sized polymeric platform [13,14]. These entities bear advantageous therapeutic index, increased solubility, lowered administration doses, and reduced side effects compared with small-molecule drugs [15]. The diversity of polymers in advanced preclinical research, the ease of conjugation of desired small-molecules and moieties, and the FDA approval of several polymer-based nanomedicines for clinical use [16,17], provide the rational to exploit polymer therapeutics for the delivery of RNAi. The idea to use polymers for the delivery of oligonucleotides (OLN) was proposed first by Behr [18] that designed cationic polyethyleneimine (PEI) to deliver DNA to several cell lines. Despite high transfection efficiency, later findings revealed high toxicity possessing an obstacle for further in vivo applications [19]. Since then, other polymers were successfully used for the delivery of OLN, amongst them are chitosan, dextran, polyaminoacids, poly(lactic-*co*-glycolic acid) (PLGA), and (*N*-(2-Hydroxypropyl)methacrylamide) HPMA copolymer [3,4]. The failure of these systems to advance towards clinical use, however, raise the need for a proper investigation and understanding of their structure–function relationship for further development of tailor-made delivery systems.

Two inherent properties of synthetic polymers possess fundamental barriers towards their clinical use: polydispersity and batch to batch irreproducibility. As synthetic polymers are built of monomers by various polymerization techniques, they are rarely monodisperse. Moreover, the exact molecular weight (*M*_w_) of each synthetic batch is hard to reproduce, as every new batch may result in different degree of polymerization (DP) [20,21]. When considering therapeutic applications, these characteristics may have fundamental consequences, as not all molecules in a heterogeneous population bear the desired activity, while they may contribute to the toxicity. The randomness of self-assembly, in addition to the polydispersity in synthesis, increases the variability among polyplexes and from batch to batch [22,23]. In fact, the first RNAi delivery vehicle that entered clinical trials, a cyclodextrin and PEG-based self-assembled delivery vehicle, might have failed to advance to bedside due to the hurdles mentioned above [9].

As a matter of cardinal importance to the field of polymer therapeutics, we set to illustrate the consequences of polymer’s length on its activity. Aminated poly(α)glutamate (PGA) was previously shown to be a potent delivery vehicle for siRNA [24,25,26,27]. In addition, it is a synthetic biodegradable polymer that is water-soluble, non-toxic, and non-immunogenic at concentrations required for activity. Amine side-chain modifications on the pending carboxylic acid moieties of PGA can serve as electrostatic binding sites for siRNA, facilitate cellular internalization via interactions with the cellular membrane leading to endocytosis [28,29], and induce endosomal escape capabilities [18]. We have previously demonstrated the in vitro silencing activity of our PGA-based aminated polymers (PGAamine) and their anti-cancer in vivo efficiency [24,25,26,27]. Here, we investigated the mechanisms dictating alterations in the function of separate synthetic batches of PGAamine, using two batches differing in their DP.

Several studies used isothermal titration calorimetry (ITC) to investigate the binding affinities between carriers and OLN. Strong affinity was correlated with stable complexes bearing enhanced in vitro and in vivo activities. However, an ideal drug delivery system confers stability during delivery while enabling site-specific release of the oligonucleotides, thus fine tuning of the affinity is required. [30,31]. Thus, we used ITC to study the thermodynamics of the complexation between our two synthetic batches of PGAamine and siRNA. Molecular dynamics (MD) simulation of the complexation process revealed structural differences, further leading to a diverse surface charge and biological stability. We therefore postulate that structural features as well as the biological stability are responsible for the variability in the silencing activity of our polyplexes. In addition, as the polymer’s *M*_w_ may directly affect the structure of the polyplexes via changing the ratio between siRNA and polymer molecules, we show that it is a crucial factor in the design of OLN delivery vehicles. Finally, we highlight the importance of adequate physico-chemical characterization of delivery systems for siRNA for optimized and precise design.

## 2. Materials and Methods

### 2.1. Materials

All chemicals and solvents were A.R. or HPLC grade. Chemical reagents were purchased from Sigma-Aldrich (Louis, MO, USA) and Merck (White House Station, NJ, USA). O-benzyl protected glutamic acid (H-Glu(OBzl)-OH) was purchased from Chem Impex (Dillon Drive, IL, USA). HPLC grade solvents were from BioLab (Jerusalem, Israel). All tissue culture reagents were purchased from Biological Industries Ltd. (Beit Haemek, Israel), unless otherwise indicated. eGFP siRNA (siCTRL), RAC1 siRNA (siRac1), Plk1 siRNA (siPlk1), and Cy5-labeled RAC1 siRNA sequences were obtained from QBI (Nez-Ziona, Israel). Long PGA was purchased from ALAMANDA^TM^ polymers (Huntsville, AL, USA).

### 2.2. Synthesis of PGAamine Polymers

#### 2.2.1. Preparation of Poly-α-glutamic Acid

The reaction system was flame dried and purged three times with Argon before use. PGA was synthesized from O-benzyl protected glutamic acid (H-Glu(OBzl)-OH) by n-carboxyanhydride (NCA) polymerization as previously published [26,32] with the following modifications: A suspension of H-Glu(OBzl)-OH (2.1 g, 8.8 mmol) in dry tetrahydrofurane (THF) (30 mL) was heated to 50 °C. (-) Limonene (1.5 mL, 6.32 mmol) was added prior to the addition of a solution of triphosgene (1.31 g, 4.4 mmol) in dry THF (10 mL). The reaction mixture was stirred under reflux for 3 h at 50 °C, under Argon atmosphere. Then, it was bubbled with Ar(g) for an additional 2 h at room temperature (RT). The resulting solution was precipitated in cold hexane, filtered, and recrystallized from a mixture of 5:3 toluene: THF by dropwise addition of cold hexane. The resulting NCA monomer was filtered (1.5 g, 5.7 mmol) and dissolved in dry dichloromethane (DCM) (30 mL). Hexylamine (1.5 μL, 0.0113 mmol) was added, and the reaction left to stir for 10 days at 7 °C, under Ar(g) atmosphere. The reaction solution was precipitated in cold ether then kept in −20 °C for 5 h. The resulting poly(γ-benzyl glutamate) (PBLG) was filtered with 0.22 μm filter. Deprotection of the γ-Benzyl was performed in 4 equivalents (per carboxylic group) of 33% HBr in acetic acid and trifluoroacetic acid (TFA) at 1:1 (*v*:*v*) ratio for 1 h. The resulting PGA was precipitated in cold diethyl ether as the free acid. The precipitate was washed with acetone and collected by centrifugation at a 50% yield. ^1^H NMR (D_2_O/NaOD; 400 MHz): δ 4.32 (1H, s), 2.36 (2H, s), 2.05, (1H, s), 1.94 (1H, s).

#### 2.2.2. Preparation of PGAamine Polymers

To a solution of PGA (50 mg, 0.38 mmol per monomer) in dry dimethylformamide (DMF) (3 mL) was added a solution of carbonyldiimidazole (CDI) (75 mg, 0.4 mmol) in dry DMF (2 mL). The reaction mixture was stirred for 1.5 h, at 25 °C, under Argon atmosphere. Tributylamine (94 µL, 0.36 mmol) was added and the reaction left to stir for 5 more min under the same conditions. A solution of Boc-ethylenediamine (0.42 mmol) in dry DMF (1.5 mL) was added and the reaction mixture was stirred for additional 3 h at the starting conditions. A solution of CDI (133 mg, 0.82 mmol) in dry DMF (1 mL) was added and the reaction mixture was stirred at 25 °C, under Argon for additional 12 h. DMF was removed under reduced pressure and the remaining oily residue was suspended in water (40 mL) and freeze-dried. Acidic deprotection of the Boc group was performed as follows: The resulting solid was dissolved in DCM (5 mL) and TFA (5 mL) was added at 0 °C. The mixture was stirred at 25 °C for 10 min then evaporated under reduced pressure. The oily residue was dissolved in double distilled water (DDW) (40 mL) and the aqueous phase was extracted with DCM (2 × 50 mL) and diethyl ether (50 mL). The aqueous phase was collected and treated with a 10% NaOH solution to reach pH = 5.5, and then freeze-dried. The remaining solid was dissolved in DDW (20 mL) and dialyzed for 48 h at 4 °C (total of 8 L of DDW). The aqueous phase was collected and freeze-dried to obtain a white powder as a TFA salt at a yield of 41%. ^1^H NMR (D_2_O; 400 MHz): δ 4.32 (1H, s), 3.45 (2H, s), 3.27 (2H, s), 2.36 (2H, s), 2.05, (1H, s), 1.94 (1H, s).

### 2.3. Physico-Chemical Characterization of PGAamine Polymers and the Obtained Polyplexes

#### 2.3.1. ^1^H-Nuclear Magnetic Resonance (NMR)

NMR spectroscopy was done using 400 MHz Avance, Bruker system (Karlshruhe, Germany).

#### 2.3.2. Multi Angle Static Light Scattering (MALS)

Molecular weight and polydispersity analysis of PGA and PGAamine polymers were performed on Agilent 1200 series HPLC system (Agilent Technologies, Santa Clara, CA, USA) equipped with a multi angle light scattering detector (Wyatt Technology Corporation, Santa Barbara, CA, USA). PGA was separated using Shodex Kw402-5F column (Showa Denko America, Inc., New York, NY, USA) and 155 mM PBS as a mobile phase, while PGAamine was separated using OHpak SB-804 HQ column (Showa Denko America, Inc., New York, NY, USA) and a mixture of 0.2 M sodium nitrate and 0.5 M acetic acid in DDW as a mobile phase. Samples were prepared at 4 mg/mL in the mobile phase buffer. Both the samples and the mobile phase were filtered using 0.022 μm filter prior to the analysis and ran at a flow 0.5 mL/min. Separation of Long PGAamine was performed using the above settings, and Kw404-4F column. A solution of Long PGAamine was prepared at a concentration of 8 mg/mL in the mobile phase, then filtered using 0.022 μm filter, loaded on the column and ran at a flow of 0.5 mL/min. The peak observed at the MALS detector was collected manually and separated to fractions.

#### 2.3.3. Electrophoretic Mobility Shift Assay (EMSA)

Evaluation of polymer:siRNA complexation at N/P ratios between 1 to 10 was performed as follows: Samples were prepared from 50 pmols of siRNA and increasing amount of PGAamine in DDW and incubated for 20 min at RT. DNA loading buffer was added to the samples, and the solution was loaded on a 2% agarose gel supplemented with ethidium bromide. A voltage of 100 volts was applied for 30 min. Sample’s run was evaluated under UV light.

#### 2.3.4. Zeta Potential Determination

Samples were prepared at PGAamine concentration of 0.06 mg/mL in 15 mM PBS. The zeta-potential measurements were performed using a PALS instrument (Wyatt Technology Corporation, Santa Barbara, CA, USA), equipped with a 532 nm laser. All measurements were performed at 25 °C using the manufacturer’s dip cell kit.

#### 2.3.5. Dynamic Light Scattering (DLS)

Samples were prepared at PGAamine concentration of 0.06 mg/mL in 155 mM PBS. DLS analysis was performed using Mobius DLS (Wyatt Technology Corporation, Santa Barbara, CA, USA), equipped with a 532 nm laser and a DLS Fluorescence Filter. Data analysis was performed according to isotropic sphere method and was measured as number averaged between 5 measurements. All measurements were performed at 25 °C.

#### 2.3.6. Transmission Electron Microscopy (TEM)

Samples were prepared at a concentration of 0.5 mg/mL of PGAamine and dropped on a formvar/carbon coated grids and stained with 2% aqueous uranyl acetate. Images were taken using JEM 1200EX TEM (JEOL Ltd., Tokyo, Japan).

#### 2.3.7. Isothermal Titration Calorimetry (ITC)

MicroCal PEAQ-ITC (Malvern Instruments Ltd., Malvern, Worcestershire, UK) was used for isothermal titration studies. One hundred and sixty-two μM PGAamine solution in 15 mM HEPES was placed in the cell, and titrated with 20 μM siRNA solution in 15 mM HEPES using a set of 18 injections of 3 μL, at 25 °C. Cumulative enthalpy exchange plots were derived from the row thermal changes, using one set of sites fitting model. A point to point control was reduced from the thermal exchange curves according to the titration of 20 μM siRNA to 15 mM HEPES. The thermodynamic parameters and binding constants were taken from the analysis done by MicroCal PEAQ-ITC analysis software (Malvern Instruments Ltd., Malvern, Worcestershire, UK). Results are representative of 3 repeats.

### 2.4. In Vitro Evaluation of Polyplexes

#### 2.4.1. Cell Culture

Human cervical carcinoma (HeLa) cells were obtained from the American Tissue Culture Collection (ATCC). HeLa cells were cultured in DMEM supplemented with 10% fetal bovine serum (FBS), 100 U/mL Penicillin, 100 µg/mL Streptomycin, 12.5 U/mL nystatin, and 2 mM L-glutamine. Cells were grown at 37 °C; 5% CO_2_.

#### 2.4.2. In Vitro Silencing Efficacy

In vitro silencing of Rac1 gene by siRac1-carrier polyplex was evaluated with psiCHECK reporter assay (Promega Madison, Fitchburg, WI, USA). One copy of a consensus target sequence of Rac1 was cloned into the multiple cloning site located downstream of the Renilla luciferase translational stop codon in the 3′-UTR region. HeLa cells (1 × 10^6^) were seeded in 10 cm dishes and were incubated in 37 °C, 5% CO_2_ for 24 h. Each cell-containing plate was transfected with 4 μg Rac1-psiCHECK™-2-based plasmids using 4 μL Lipofectamine^®^ 2000 (Life Technologies, Grand Island, NY, USA). Following 24 h, cells were reseeded in 96-well plate at final concentration of 4000 cells per well and incubated overnight. Cells expressing Rac1 siRNA reporter plasmid were transfected with siRac1 or siCTRL either complexed with PGA cationic carrier (at 100, 250, or 500 nM siRNA) or with Lipofectamine^®^ 2000 (at 50 nM siRNA) as a control or left untreated. Following 72 h, medium was completely removed from cells and the cells were lysed for 20–30 min in room temperature in gentle rocking by the addition of 50 µL/well Luciferase lysis solution. Renilla and Firefly Luciferase activities were measured in each of the wells of the 96 wells plate, using Dual-Luciferase^®^ Assay kit (Promega, Madison, WI, USA) according to manufacturer procedure. Aliquots of 15 µL of cell lysate from each sample were transferred to a 96-well white plate. Luciferase substrate (LARII, 40 µL) was added to each extract and Firefly Luciferase activity was measured by luminescence microplate Reader (Mithras LB 940 Multimode Microplate Reader, Berthold Technologies, Bad Wildbad, Germany), then 40 µL of Stop&Glo Reagent was added to each of the samples and Renilla Luciferase activity was measured immediately afterwards. The Renilla luciferase activity is expressed as the percentage of the normalized activity value (Renilla luciferase/Firefly luciferase) in the tested sample relative to the normalized value obtained in cells transfected with the corresponding psiCHECK™-2 plasmid only (without siRNA nor polyplex).

#### 2.4.3. Cell Viability Assay

HeLa cells were seeded and treated with polyplex as described above in the section entitled in vitro silencing efficacy. Following 72 h, the number of viable cells was assessed by 3-(4,5-dimethylthiazol-2-yl)-2,5-diphenyltetrazolinium bromide (MTT) assay. Thirty μL of 3 mg/mL MTT solution in PBS were added to the wells and incubated for 4 h. The medium was then replaced by 200 μL of dimethyl sulfoxide (DMSO) to dissolve the formazan crystals formed, and incubated for 20 min at 37 °C. Absorbance of the solution was measured at 560 nm by SpectraMax^®^ M5 plate reader (Molecular Devices, Sunnyvale, CA, USA). Percent of viable cells was normalized to the viability of non-treated cells (100% viability).

#### 2.4.4. Western Blot Analysis

HeLa cells were seeded in 10 cm plates at a density of 500,000 cells/plate. After 24 h, cells were treated with 500 nM of siPlk1 complexed with either Short or Long PGAamine, alone. Following 48 h, cells were harvested and lysed using NP40 reagent. Lysates ran on an acryl amide gel under 120 V for ~2 h. Gels were transferred to nitrocellulose membrane under 80 mA current over-night. Membrane was blocked with 5% skim milk for 1 h, reacted with rabbit anti-Plk1 antibody (Cell Signaling Technology, Danvers, MA, USA) (1:500 in TBST) and mouse anti-HSC 70 antibody (Santa Cruz Biotechnology, Dallas, TX, USA) (1:40,000) over-night at 4 °C, and then with horseradish peroxidase (HRP)-conjugated goat anti-rabbit and goat anti-mouse secondary antibodies (Jackson Immunoresearch, Baltimore, PA, USA and abcam, Cambridge, MA, USA), both at 1:10,000 in TBST for 1 h. Blots were developed using Wester Supernova ECL kit (Cyanagen, Bolonga, Italy) according to the manufacturer’s protocol.

#### 2.4.5. Wound Healing Assay

Inhibition of wound closure was studied using IncuCyte ZOOM^®^ Live Cell Imaging system (Essen BioScience, Ann Arbor, MI, USA). HeLa cells were seeded in 96-well ImageLock tissue culture plate (35,000 cells/well) in DMEM supplemented with 10% FBS, 2 mM L-glutamine and were allowed to grow for 12 h (37 °C; 5% CO_2_). Then, cells were treated with 500 nM of siPlk1 complexed with either Short or Long PGAamine for 40 h. A wound was created in each well using a 96-pin wound-making tool (WoundMaker™, Essen BioScience, Ann Arbor, MI, USA), the wells were washed and added with fresh treatments. To image wound confluence over time, the plate was placed in the IncuCyte ZOOM™ incubator and phase contrast images were taken at 30 min intervals over a course of 140 h using 10× objective. Results were calculated by the IncuCyte™ Software and presented as percentage of wound confluence compared with untreated cells.

#### 2.4.6. Intracellular Trafficking Study

HeLa cells were seeded (50,000 cells/well) on 13 mm cover glasses in 24 wells plate and incubated for 24 h. The cells were treated with 100 nM siRNA-equivalent concentration alone or complexed with Short/Long PGAamine for 1.5, 4, and 24 h. The cells were washed several times with PBS, fixed with 4% paraformaldehyde for 30 min at room temperature and washed with PBS again. Cells were then permeabilized by incubation in Triton X-100 0.2% solution in PBS for 10 min, followed by blocking in a solution of 1:100 normal mouse IgG1 and normal rabbit IgG (Santa Cruz, Heidelberg, Germany). Cells were stained with mouse anti-EEA1 (BD, East Rutherford, NJ, USA) and with rabbit anti-LAMP1 (Cell Signaling Technology, Danvers, MA, USA) primary antibodies, and then with Goat anti mouse IgG-FITC and Goat anti rabbit IgG-Rhodamine secondary antibodies (Santa Cruz, Heidelberg, Germany). Intracellular trafficking was followed using Leica SP8 confocal imaging systems (×60 Magnification) (Leica Microsystems, Wetzlar, Germany), and colocalization was assessed using the Imaris software (Bitplane, Zurich, Switzerland). Averages and standard deviations (SD) were calculated based on at least 5 fields.

### 2.5. Molecular Dynamics (MD) Simulation

MD simulation was performed using HyperChem 8.06 software (Hypercube, Inc. Gainesville, FL, USA). Rac1 siRNA sequence, Long PGAamine containing 56 units, and Short PGAamine containing 27 units, were each separately energy minimized by geometrical optimization using the CHARMM27 force field, the Polak–Ribiere algorithm, and convergence parameter of 0.1 cal/mol. Then, two simulations were run separately: a simulation of 3 molecules of Long PGAamine and siRNA, and a simulation of 7 molecules of Short PGAamine and siRNA. In both simulations, the PGAamine polymers were placed near the periphery of the siRNA molecule at a reasonable distribution, following by geometry optimization using convergence parameter of 0.1 cal/mol, the CHARMM27 force field, and the Polak–Ribiere algorithm. Following energy minimization, MD simulation was performed for 100 ps, in steps of 0.0005 ps, at a constant temperature of 300 K and bath relaxation time of 0.1 ps, using CHARMM27 force field. All stages were performed in vacuum.

### 2.6. Biostability and Toxicity Assays

#### 2.6.1. Plasma Stability

Polyplexes prepared at 50 pmol siRNA/samples were incubated in 70% full mouse plasma for up to 27 h. The polyplexes were then loaded on a 2% agarose gel supplemented with ethidium bromide that was further supplied with 100 V for 15 min. Migration extant was validated under UV light.

#### 2.6.2. Heparin Displacement Assay

Displacement of siRNA by the polyanion heparin was assessed using agarose gel. PGAamine:siRNA polyplexes were prepared to 50 pmol siRNA per sample, then incubated with 0.01–0.2 heparin international units (IU) for 15 min. DNA loading buffer was added to the samples, and the samples were loaded on a 2% agarose gel supplemented with ethidium bromide. A voltage of 100 volts was applied for 15 min. Migration extant was validated under UV light.

#### 2.6.3. Red Blood Cell Lysis

Polyplexes were prepared at siRNA concentrations that equivalent to 500 nM (in vitro dose) or 8 mg/kg (0.367 mg/mL polymer, in vivo dose). SDS (positive control) and 70 KDa dextran (negative control) solutions were prepared at similar concentrations. Samples were incubated for 1 h at 37 °C in equal volume of 2% (*w*/*w*) C57 mouse red blood cells (RBC) solution that was withdrawn from two mice. Samples were centrifuged at 1000 RCF for 10 min. The hemoglobin containing supernatants were then transferred to a 96 wells plate and absorbance was measured at 550 nm using a SpectraMax M5e plate reader (Molecular Devices, Sunnyvale, California, USA). Results were normalized to 100% lysis obtained by incubation of RBC in 1% (*v*/*v*) Triton X-100.

### 2.7. Statistical Analysis

Data were presented as means ± standard deviation (represented graphically as error bars). Statistical significance was determined using *t*-test. *p* < 0.05 was considered statistically significant.

## 3. Results

### 3.1. Synthesis and Characterization of Long and Short Aminated Poly(α)glutamates

**Scheme 1:**
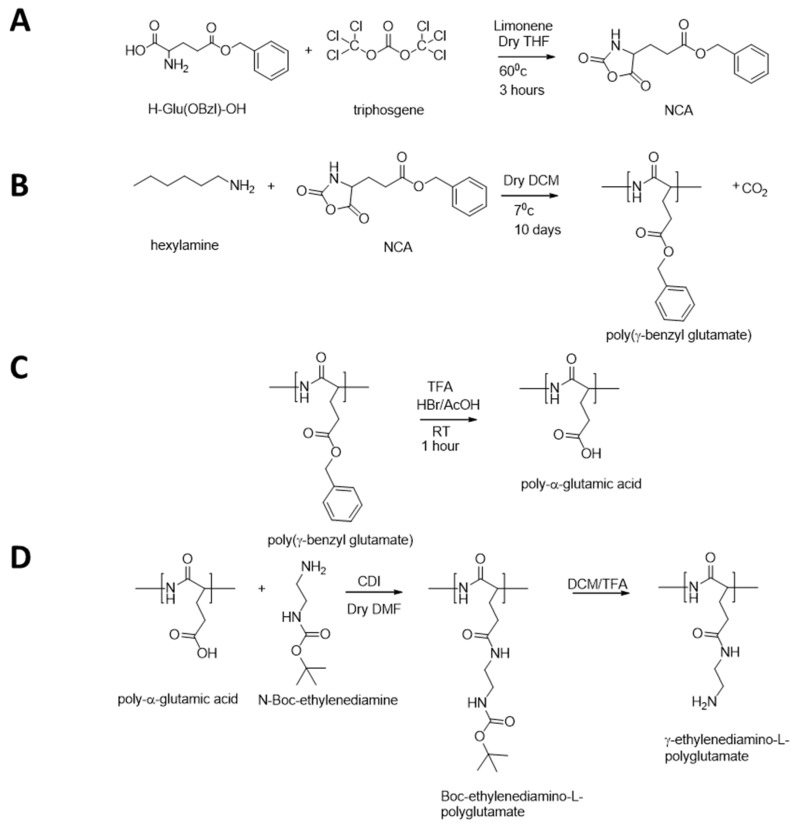
Synthesis of PGAamine: (**A**) Synthesis of γ-Benzyl L-glutamate α-*N*-carboxyanhydride (NCA) from H-Glu(Obz)-OH; (**B**) Polymerization of the NCAs using hexylamine initiator; (**C**) Acidic deprotection of the γ-benzyl; (**D**) Conjugation of *N*-Boc-ethylenediamine moieties followed by acidic deprotection of Boc.

**Figure 1:**
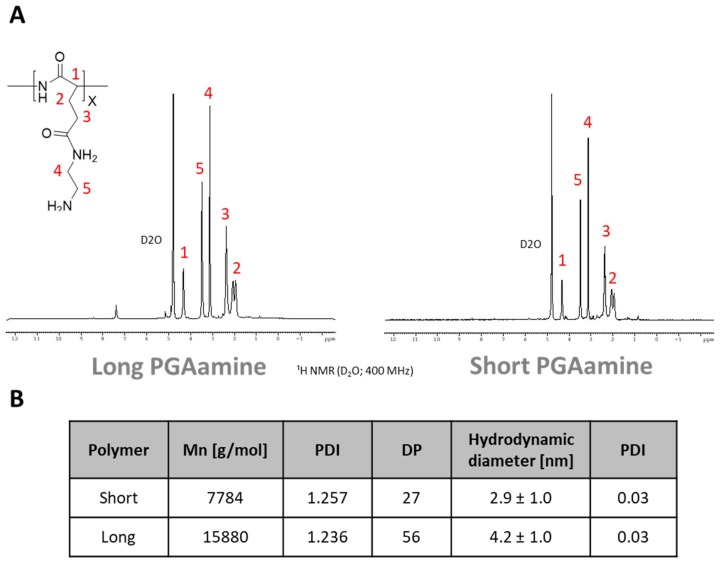
Characterization of Long and Short PGAamine polymers. (**A**) ^1^H NMR spectra obtained at 400 MHz; (**B**) a table summarizing the Molecular weight (*M*_n_), polydispersity index (PDI), and calculated degree of polymerization (DP) as obtained by MALS, hydrodynamic diameter, and polydispersity index (PDI) as obtained by Mobius DLS.

Short Poly(α)glutamate (PGA) was synthesized from O-benzyl protected glutamic acid (H-Glu(OBzl)-OH) as was previously described [26,32] (Scheme 1A–C): γ-Benzyl l-glutamate α-*N*-carboxyanhydride (NCA) was synthesized from γ-Benzyl L-glutamate (H-l-Glu(OBzl)-OH) using triphosgene. Hexylamine served as an initiator to polymerize the α-*N*-carboxyanhydrides (NCAs) by ring opening polymerization, following by acidic deprotection of the γ-Benzyl group. Long and Short PGA precursors were characterized by ^1^H-NMR and MALS (Figure S1). PGA at the *M*_w_ (*M*_n_) of 11,820 g/mol was designated as “Short”, and calculated to bear 92 glutamate units, while PGA of 21,020 g/mol was designated as “Long” and was calculated to bear 163 glutamate units. *N*-Boc-ethylenediamine was conjugated to the PGA precursors via the pending carboxylic acid moieties using carbonyldiimidazole (CDI) conjugation reagent, following by acidic deprotection to remove the Boc group, as was published before [26] (Scheme 1D). ^1^H-NMR spectra demonstrated 100% amination (Figure 1A), that was obtained using 3 eq. of CDI and 1.1 eq. of *N*-Boc-ethylenediamine. The obtained Long and Short PGAamine polymers were characterized by MALS to bear *M*_w_ (*M*_n_) of 15,880 and 7784 g/mol respectively, corresponding to DP of 56 and 27 units (Figure 1B). The Long and Short PGAamine polymers were further characterized for their hydrodynamic diameters in physiological solution (PBS), using dynamic light scattering (DLS) (Figure 1B). Long PGAamine obtained size of 4.2 ± 1.0 nm, whilst the short PGAamine demonstrated slightly smaller size (2.9 ± 1.0 nm), both polyplexes demonstrated a narrow polydispersity index (PDI) of 0.003.

### 3.2. Physico-Chemical Characterization of the Polyplexes Formed by Electrostatic-Based Complexation of Long or Short PGAamine with siRNA

**Figure 2:**
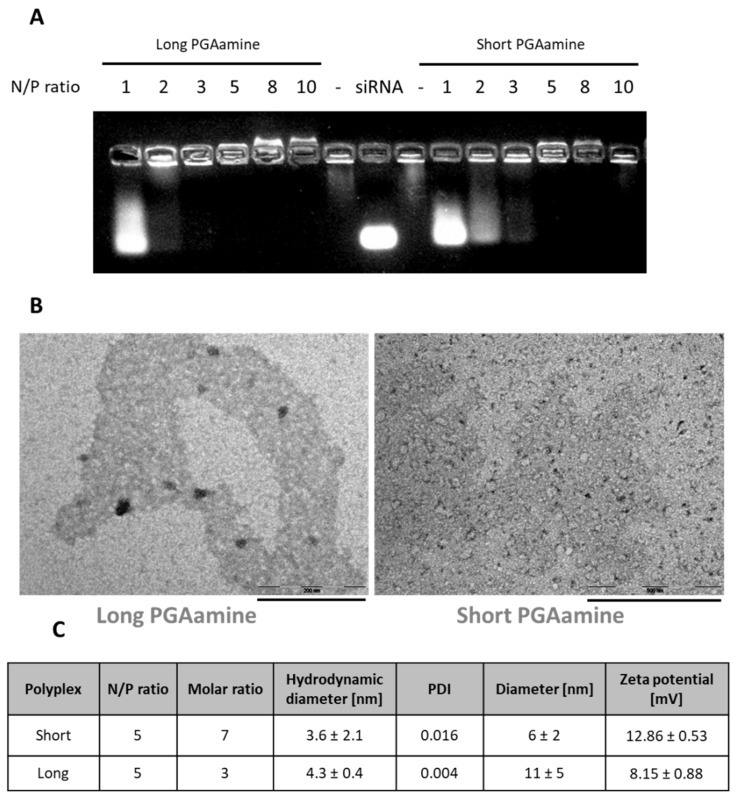
Characterization of Long and Short PGAamine:siRNA polyplexes. (**A**) Electrophoretic Mobility Shift Assay (EMSA) of PGAamine complexed with siRNA at increasing N/P ratios; (**B**) TEM images of Short and Long PGAamine polyplexes complexed with siRNA at N/P ratio of 5 (scale bars: Long—200 nm, Short—500 nm); (**C**) A table summarizing the characteristics of the selected PGAamine:siRNA polyplexes: their N/P and molar ratios, their hydrodynamic diameters, and polydispersity indexes (PDIs) as obtained by Mobius DLS, their average diameter as obtained by TEM, and their zeta potentials as obtained by PALS.

Polyaminated polymers bear net positive charge in physiological pH, enabling the formation of electrostatic-based complexation with the negatively charged siRNA. To validate the ability of PGAamine polymers to bind siRNA, EMSA was applied (Figure 2A). siRNA (50 pmols) were incubated with either Long or Short PGAamine, at various terminal-nitrogen to phosphate (N/P) ratios, and loaded on an agarose gel stained with ethidium bromide. The samples were allowed to migrate under voltage-supply. Free siRNA migrated towards the anode due to its negative charge, while retardation of migration was observed with positive polymer due to charge-neutralization of siRNA in resulting complex. Though both Long and Short PGAamine polymers complexed with siRNA, the polymers differed in their migration pattern: Short PGAamine required higher N/P ratio for full neutralization of siRNA charge, demonstrating complete retardation of migration only at N/P ratio of 5, while Long PGAamine neutralized the charged siRNA already at N/P ratio of 3. Successful transfection of cancer cells with cationic polyplexes is traditionally attributed to a positive surface charge, enabling electrostatic attachment to negatively charged components of the cellular membrane, followed by endocytosis [33]. Nevertheless, a high positive charge is known to be toxic and should be avoided for intravenous (IV) administration [34]. Postulating that N/P ratio of 5 will confer cellular internalization capabilities alongside low toxicity to our polyplex, it was selected for further experimentation and physicochemical characterization (Figure 2B,C). This N/P ratio, however, translated to different molar ratios that were used for the mixing of either Long or Short PGAamine polymers with siRNA (7 and 3, respectively). Interestingly, although both polyplexes demonstrated positive zeta potential, Short PGAamine:siRNA polyplex had a higher zeta potential than the Long one (12.86 ± 0.53 mV compared with 8.15 ± 0.88 mV). To further elucidate the size and morphology of Long and Short PGAamine:siRNA polyplexes, DLS and TEM were performed. The hydrodynamic diameters of our polyplexes, formed at low concentrations (0.06 mg of polymer per mL) in physiological solution (PBS, pH 7.4), were almost similar (3.6 ± 2.1 nm and 4.3 ± 0.4 nm for the Short and Long polyplexes respectively). TEM images confirmed the sizes of Short and Long PGAamine:siRNA polyplexes, demonstrating diameters of 6 ± 2 nm and 11 ± 5 nm, respectively.

### 3.3. Isothermal Titration Calorimetry (ITC)

**Figure 3:**
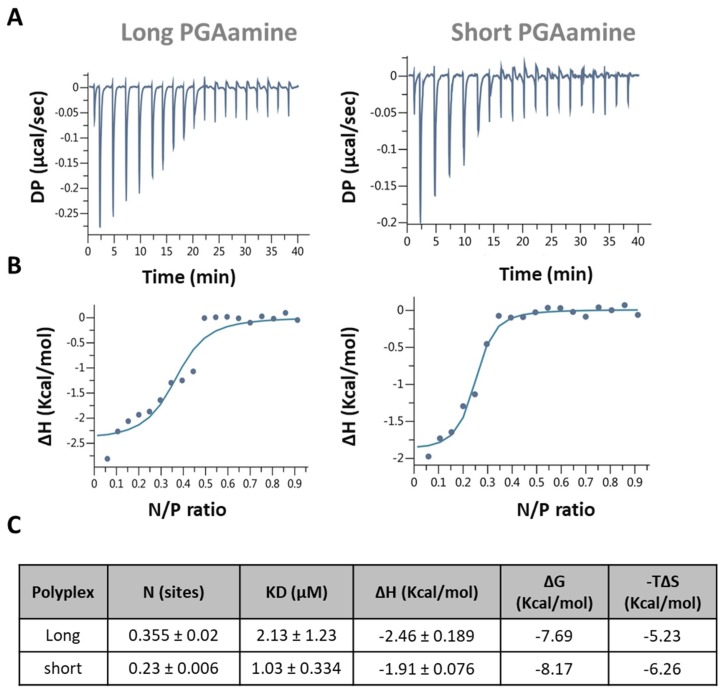
Isothermal titration calorimetry (ITC) of the binding between PGAamine polymers and siRNA. (**A**) The row data was obtained from a titration of 20 μM siRNA solution (760 μM Phosphate groups) into Short or Long PGAamine solution containing 81 μM aminated glutamate units, in 15 mM HEPES; (**B**) Cumulative enthalpy exchange plotted against increasing N/P ratios; (**C**) The thermodynamic parameters derived from the cumulative enthalpy exchange plots.

The binding affinity between a cationic carrier and siRNA might have a crucial impact on the silencing activity. Strong affinity confers protection of the siRNA via delivery allowing accumulation in the target site and enhanced silencing activity. However, fine-tuning between high and low affinity should be taken into consideration to enable release of the OLN in the target site [22,35]. The *M*_w_ of the polymeric delivery vehicle was shown before to affect the binding to the cargo, as low *M*_w_ methoxy-poly(ethylene glycol)s-cholane (mPEG-cholane) demonstrated a higher binding affinity with the recombinant human growth hormone (rh-GH), compared with high *M*_w_ mPEG-cholanes [36]. In accordance, the binding affinity between low *M*_w_ Chitosan and siRNA was slightly higher than that of the high *M*_w_ Chitosan [37]. The effect of binding affinity on the efficacy was demonstrated by Chou et al., who showed that adding lysine-rich domains to poly-histidine resulted in increased binding affinity to siRNA, further reflected in enhanced silencing activity [31]. To study the nature of the binding interactions between PGAamine and siRNA, ITC was performed (Figure 3). siRNA solution (20 μM, 760 μM single nucleotides) was loaded on the syringe and added stepwise to a cell containing 162 μM aminated glutamate units polymerized in either Short or Long PGAamine polymers. Each injection resulted in thermal exchange, activating a feedback loop to reserve a constant temperature according to a reference cell (Figure 3A—upper panel). The area under the peaks was integrated and fitted to give the cumulative enthalpy changes versus the N/P ratio using one set of sites fitting model (Figure 3A—lower panel). A point-by-point control was subtracted from the thermal exchange curves according to the titration of siRNA into the buffer (Figure S2). The initial interaction of polymer and siRNA was exothermic in both polymer types, demonstrating Δ*H* values of −2.46 ± 0.189 and −1.91 ± 0.076 Kcal/mol for the Long and Short polymers, respectively, that are typical of the interactions of cationic polymers and OLN [30] (Figure 3C). Such exothermic reaction was attributed before to non-ionic interactions of polyaminated polymers and siRNA, possibly due to the formation of hydrogen bonds [31]. In both curves, the exothermic reaction reached a plateau when all strongly-binding sites on the polymers were occupied, starting a second region of interaction which is characterized by a constant heat omitted with each injection of siRNA. This region is attributed to the dilution of siRNA in buffer [38,39,40], as indicated by the similarity to heat exchanges resulting from the titration of siRNA into HEPES (Figure S2). The constants of binding dissociation, derived from the fitting plots, were in the micromolar range for each binding unit, but summed to a nanomolar range relating to an entire polymer-siRNA binding, indicating high-affinity between PGAamine polymers and siRNA. In agreement with previous studies, the Short PGAamine demonstrated a non-significant higher binding affinity with siRNA [36,37] compared with the Long PGAamine. The stoichiometry of strong association was 0.355 and 0.230 for the Long and Short PGAamines, respectively, implying that further binding between the polymers and siRNA occurs with different thermodynamic behavior, at lower affinities and higher concentrations. Such a multimodal binding behavior between cationic polymers and OLN was demonstrated before by the binding of poly(glycoamidoamine)s and plasmid DNA (pDNA) [39], polyethyleneimine (PEI) and DNA [40], and polyaminoacids and siRNA [31]. Interestingly, the entropy exchange was higher for the binding of short PGAamine and siRNA (−*T*Δ*S* values of −5.23 and −6.26 Kcal/mol for the Long and Short polymers, respectively), indicating that more disruption of counter ions and the hydration layer was taking place in the association of Short PGAamine and siRNA compared with the association of Long PGAamine and siRNA. Such an entropically driven endothermic interaction was associated before with the displacement of counter ions and hydration interactions due to binding of cationic polymers and OLN [31]. Favored enthalpies and entropies in the interactions of both polymers with siRNA dragged the complexation reaction to occur spontaneously, as reflected by Δ*G* values of −7.69 and −8.17 Kcal/mol for the Long and Short polymers, respectively. These values are in good agreement with the findings of Sheikhi Mehrabadi et al., who used MD simulation to calculate the effective free energy of binding between cationic dendrimers and dsDNA. The individual contribution of each amine moiety modified on the surface of a polyglycerol-cored dendrimer ranged between −7.0 to −12.2 Kcal/mol [41]. In addition, another MD simulation calculated the normalized interactions between poly(amidoamine) (PAMAM) dendrimers and dsRNA to give free energies of −5.0 to −17.0 Kcal/mol [42].

### 3.4. Long PGAamine:siRNA Polyplexes Demonstrated Silencing Activity and Anticancer Efficacy in HeLa Cells

**Figure 4:**
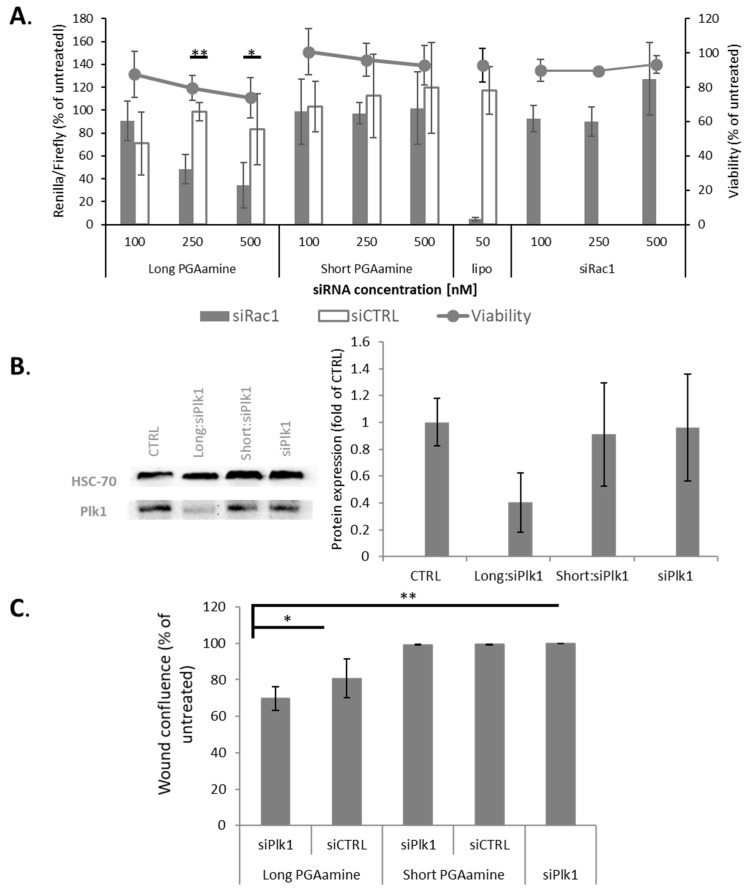
In vitro activity of Long and Short PGAamine:siRNA polyplexes in HeLa cells. (**A**) Silencing activity, as measured by psiCHECK reporter assay (bars), and viability, as measured by MTT (circles). (**B**) Plk1 protein expression 48 h following treatment with 500 nM siplk1 complexed with Long or Short PGAamine; Left panel—a representative gel, right panel—quantification of 3 repeats; (**C**) Wound confluence following 140 h from scratch and 180 h from treatment with PGAamine:siPlk1 polyplexes. Data represents mean ± SD. Statistical significance was determined using *t*-test, significance: * *p* < 0.05, ** *p* < 0.005.

RNAi has emerged as a promising anticancer therapeutic approach due to its ability to silence undruggable oncogenes [3,43]. To evaluate the silencing activity of our polyplexes consisting of either Long or Short PGAamine and siRNA on human HeLa cervical carcinoma cells, we investigated the Rac1 expression using a psiCHECK reporter assay. The small GTPase protein Rac1 is a known regulator of cellular motility that is associated with migration, metastasis, and invasiveness of various cancers [44,45]. Rac1′s expression, however, is not directly connected to cellular death [46], therefore, we have selected siRac1 to study gene silencing, while avoiding the cellular killing effect. PsiCHECK plasmid-transfected HeLa cells were treated with either Long or Short PGAamine complexed with siRac1 (Figure 4A bars). While Short PGAamine:siRac1 polyplexes did not silence the target mRNA, Long PGAamine:siRac1 polyplexes silenced mRNA expression to 48% and 34% compared to untreated cells, when administered at concentrations of 250 and 500 nM (siRNA dose), respectively. As expected, both free siRac1 and control siRNA did not silence the target mRNA, regardless of the presence and size of the carrying polymer. The commercial transfection reagent, Lipofectamine^®^ 2000, served as a positive control and demonstrated high and selective silencing activity of more than 95% compared with untreated cells. The cellular toxicity of our treatments was evaluated using MTT assay (Figure 4A circles). Both polymers were non-toxic to cells, retaining more than 74% viability, with slightly higher toxicity demonstrated by the Long polyplex. 93% viability was shown following treatment with 50 nM siRNA complexed with Lipofectamine^®^ 2000. To further validate the size-dependent activity of PGAamine polymers, various synthetic batches of PGAamine were analyzed for their molecular weights by MALS, and for their silencing activity using psiCHECK reporter assay. Results are summarized in Figure S3A. Two active polymer batches were synthesized, and both were comparably long (15,880 g/mol and 20,200 g/mol). Six other non-active polymer batches were shorter, and bared molecular weights ranging from 6476 to 11,060 g/mol. Figure S3B shows the silencing activity of PGAamine batch #6 that bears a molecular weight of 20,200 g/mol and was found active in the psiCHECK reporter assay. Specific mRNA silencing of around 54% was demonstrated using PGAamine #6:siRac1 polyplexes at a concentration of 500 nM, while PGAamine #6:siCTRL polyplexes did not cause mRNA silencing. To further demonstrate the size-dependent silencing activity of our polymers, and to rule out effects of batch-to-batch variability, Long PGAamine was separated into fractions (Figure S4A). These fractions were further analyzed for their molecular weights by MALS and categorized into shorter and longer fractions, bearing molecular weights of 12,180 and 15,440 g/mol, respectively. As expected, both fractions had much lower PDI compared with that of the original Long PGAamine (1.082 and 1.021 compared with 1.236). The silencing activity of these fractions was tested by psiCHECK reporter assay (Figure S4B). While the longer fraction demonstrated silencing activity of 55% and 72% when complexed with siRNA at concentrations of 250 and 500 nM, respectively, the shorter fraction displayed less than 35% silencing activity at siRNA concentration of 500 nM. Plk1 is an established proto-oncogene known to promote mitosis. Its overexpression was demonstrated in various cancers, associated with low survival and poor prognoses. Plk1′s activity was linked with enhanced metastasis, migration, and invasiveness, rendering Plk1 an attractive target for anti-cancer therapy [47,48,49,50,51]. We have previously shown that by knocking down (KD) the expression of Plk1, we were able to decrease tumor volume and prolong the survival of mice bearing orthotopic ovarian tumors [52]. Lately, we have demonstrated that a combination of Plk1 inhibition together with miR-34a expression was an effective anticancer strategy in PDAC mouse model [25]. To demonstrate the effect of the polymer’s *M*_w_ on the functional inhibition of genes, HeLa cells were treated with either Long or Short PGAamine polyplexed with siPlk1. Western blot demonstrated silencing of the Plk1 protein by Long PGAamine:siPlk1 polyplexes, but not by Short PGAamine:siPlk1 polyplexes, while siPlk1 alone did not downregulate the expression of the Plk1 protein (Figure 4B). In accordance, Long PGAamine:siPlk1 polyplexes significantly inhibited wound closure following 180 h from treatment, demonstrating 30% reduced confluence of the wound compared with treatment with siPlk1 alone, while Short PGAamine complexed with either siPlk1 or with siCTRL was unable to inhibit the wound closure (Figure 4C). Long PGAamine:siCTRL polyplexes demonstrated a nonspecific anti-migratory effect to a lesser extent than Long PGAamine:siPlk1 polyplexes, showing ~20% reduced wound confluence compared with siPlk1 treatment.

### 3.5. Long and Short PGAamine Polyplexes Demonstrated Similar Colocalization Ratios with Endocytic Markers

**Figure 5:**
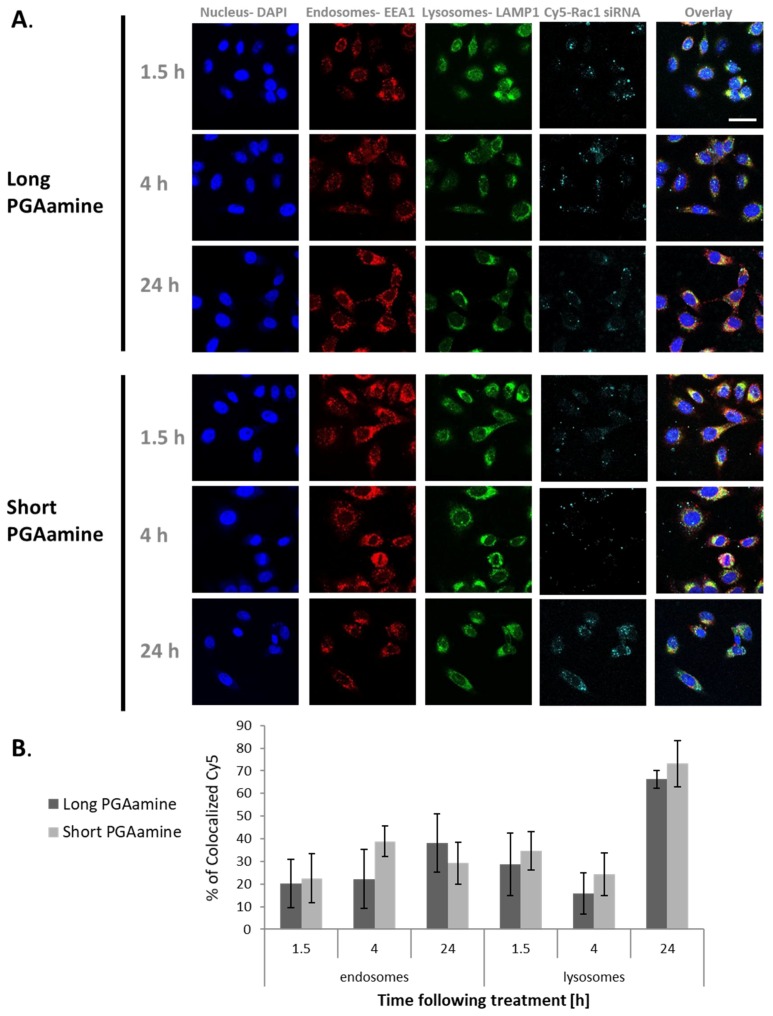
Colocalization ratios of Long and Short PGAamine polyplexes with endolysosomal markers. (**A**) Long and Short PGAamine polymers were complexed with Cy5-Rac1 siRNA and applied to HeLa cells at 100 nM siRNA dose. Representative confocal images at 1.5, 4, and 24 h following treatment are shown. Scale bar = 20 μm; (**B**) Quantification indicates similar colocalization of the polyplexes with endosomes and lysosomes. Data represent mean ± SD.

The internalization route and intracellular trafficking pathways of complexes composed of cationic polymers and siRNA fundamentally affect their silencing activity. Recent studies show that all 3 types of pinocytosis, namely macropinocytosis, clathrin-mediated endocytosis (CME), and caveolae-mediated endocytosis (CvME), were associated with the internalization of cationic polyplexes to HeLa cells [53,54]. However, not all trafficking pathways result in similar silencing activity: various researches show that CME has a minor contribution to successful gene silencing compared with other internalization pathways [55,56]. Thus, we have tested the hypothesis that distinct internalization mechanisms govern the internalization of Long and Short PGAamine polyplexes to HeLa cells, resulting in lower silencing activity of the Short PGAamine:siRNA polyplexes. Rac1-siRNA was conjugated to Cy5, and applied to the cells at a concentration of 100 nM, carried either by Long or Short PGAamine. The cells were fixed after 1.5 h, 4 h, or 24 h, and stained for the endolysosomal pathway organelles using the endosomes and lysosomes markers EEA1 and LAMP-1, respectively (Figure 5A). Notably, both Short and Long PGAamine polymers were able to facilitate the internalization of Cy5-Rac1 siRNA to the cells, while Cy5-siRNA alone hardly crosses the cellular membrane even after 24 h from the treatment (Figure S5). Moreover, both polymers demonstrated similar colocalization ratios with the endolysosomal pathway markers (Figure 5B). In accordance with previous studies, both polyplexes colocalized at 20–40% with endosomes up to 24 h from treatment, and demonstrated time-dependent accumulation in lysosomes [25,26], although the ~30% of siRNA not colocalized with lysosomes was still available to induce gene silencing.

### 3.6. Computerized Model of Long and Short PGAamine Binding with siRNA

**Figure 6:**
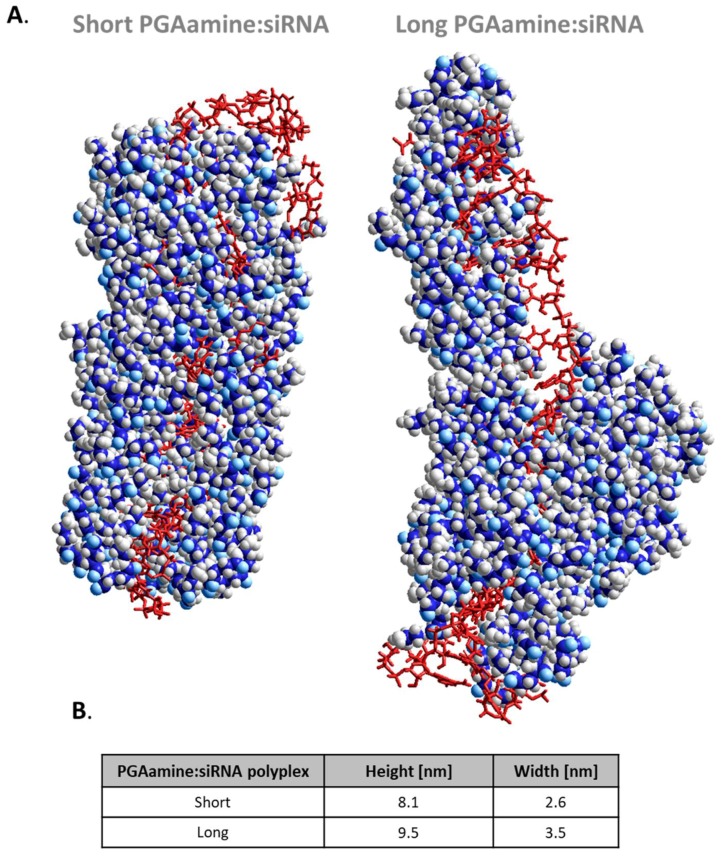
Molecular Dynamics (MD) simulation of complexation between PGAamine and siRNA. (**A**) The structures of Long and Short PGAamine polyplexes as obtained at the final time point (100 ps) of molecular dynamics simulation. The siRNA is shown in red as tubes, whereas the Long (3) and Short (7) polymers are shown in blue and grey as balls; (**B**) A table summarizing the distance between the furthest atoms as measured from top to bottom and from left to right, on the final structures of polyplexes as obtained by MD simulation.

Postulating that the different silencing activities of Long and Short PGAamine polyplexes might stem from different structural characteristics, MD simulation was performed. The simulation for Long PGAamine included 3 Long polymers and one siRNA molecule, while the simulation of Short PGAamine included 7 Short polymers and one siRNA molecule in accordance with the molar ratios (Figure 2C). The polymers were initially placed at the proximity of the siRNA periphery at a reasonable distribution and allowed to complex under RT (300 K) for 100 ps (Supplementary movies). The CHARMM27 force field was used, as it is suitable for OLN and proteins, and implicitly considers aqueous surroundings via a distance-dependent dielectric constant [57]. For both simulations, complexes were obtained following ~50 ps, while after ~50 ps of MD simulation only minor alterations in the coordination of atoms occurred, and the main structures did not change. The complexes formed composed the entire components included in the simulation (Figure 6), demonstrating the feasibility of such assembly, and approving our hypothesis regarding possible additional binding sites to those ascribed to the sharp ascending region of the ITC cumulative plot (Figure 3). Importantly, these complexes reflect an assembly that is purely based on a stoichiometric ratio of 5 N/P, and does not consider aggregation effects and supramolecular arrangements that occur at higher concentrations. The sizes of the polyplexes could be measured from the final structure of MD simulation, to obtain heights of 8–9 nm and widths of 2.5–3.5 nm for the Short and Long polyplexes, respectively (Figure 6B). These sizes are in good agreement with the sizes obtained by DLS and TEM measurements (Figure 2B,C). The cardinal structural difference between the two complexes, as shown by the final structures of MD simulation, was the siRNA coverage by PGAamine polymers: while Short PGAamine polymers completely covered the siRNA, Long PGAamine left a portion of the siRNA backbone exposed to the surroundings. This naked portion was probably not subjected to counter ions and hydration layer disruption via the complexation process, possibly explaining the lower enthalpy exchange associated with the binding of Long PGAamine and siRNA compared with Short PGAamine, as was measured by ITC (Figure 3C). These structural characteristics are further reflected in the different zeta potential of the complex (Figure 2C), as the short PGAamine complex demonstrated higher zeta potential, exposing mostly the protonatable amine-moieties to the outer surroundings. This difference might have a prominent effect on the stability of the polyplexes in physiological fluids, as it is known that positive particles tend to attract macrophages and rapidly degrade in the bloodstream [23]. On the other hand, the availability of the siRNA sequence to the surroundings, while still protected by the carrier, demonstrated by the structure of Long PGAamine polyplexes, may improve its biological activity.

### 3.7. The Biological Stability and Toxicity of Long and Short PGAamine:siRNA Polyplexes

**Figure 7:**
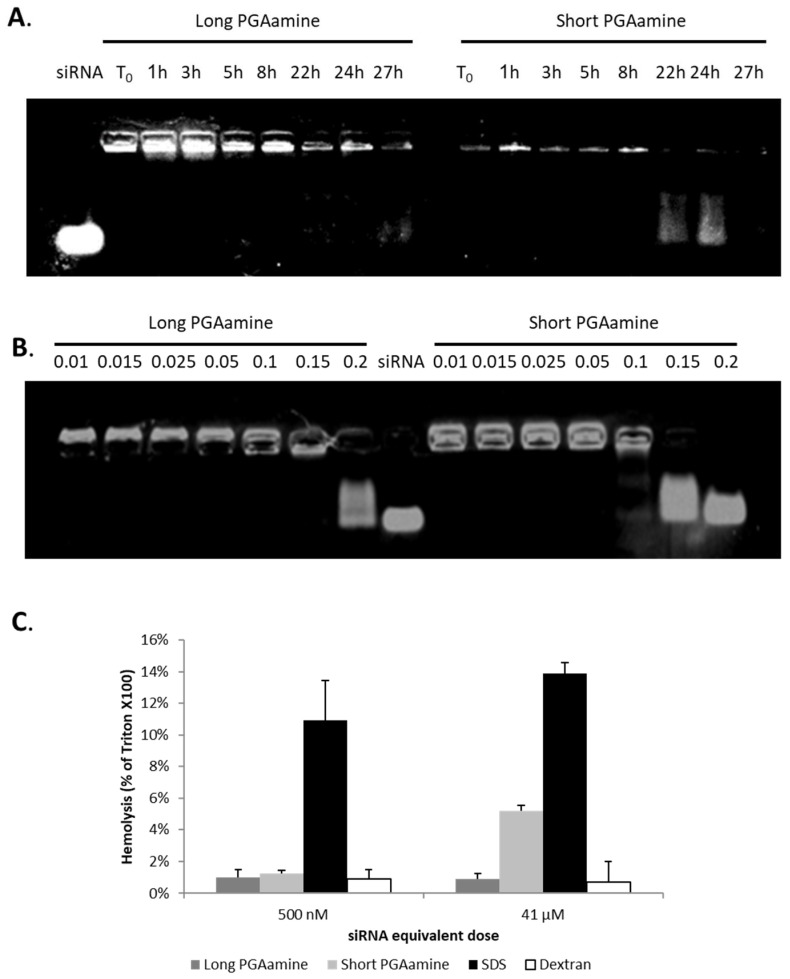
The biological stability and toxicity of Long and Short PGAamine:siRNA polyplexes. (**A**) Stability of polyplexes following incubation in 70% mouse plasma; (**B**) Replacement of siRNA by heparin, the international units (IU) of heparin per 50 pmol of siRNA specified above the gel image; (**C**) Red blood cell (RBC) lysis after 1 h incubation with either Long or Short PGAamine polyplexes. Data represents mean ± SD.

To further investigate the mechanisms governing the different silencing activities of Long and Short PGAamine when carrying siRNA, the biological interactions between our polyplexes and physiological fluids were examined. Polyplexes composed of either Long or Short PGAamine and siRNA at N/P ratio of 5, were incubated in 70% full mouse plasma for up to 27 h (Figure 7A). Samples were loaded on an agarose gel and imaged to evaluate the migration of free siRNA following complex degradation. As opposed to the non-significant different affinities measured by ITC (Figure 3B), the biological stabilities of the polyplexes differed: While Long PGAamine:siRNA polyplexes were stable for up to 24 h and started to degrade only after 27 h of incubation, Short PGAamine:siRNA polyplexes demonstrated lower stability in plasma, and started to degrade and release siRNA already following 22 h of incubation. By 27 h, there were no complexes of Short PGAamine and siRNA left. To establish the differences in the biological stability of the polyplexes, heparin displacement assay was performed. Previous studies demonstrated the importance of optimal binding strength based on heparin displacement studies [35]. We demonstrate the replacement of siRNA by the addition of 0.2 heparin IU to polyplexes of Long PGAamine and siRNA, while it required only 0.15 heparin IU to replace the same amount of siRNA (50 pmol) when bound to Short PGAamine (Figure 7B). These results further validated that Short PGAamine provided lower protection to siRNA at a biological scenario, compared with Long PGAamine. This lower stability further reflected in lower activity, possibly due to reduced siRNA dose available to induce gene silencing. To further evaluate the potential of our polyplexes for systemic delivery, an ex vivo RBC lysis assay was applied (Figure 6C). Samples were prepared at concentrations corresponding to the in vitro effective siRNA concentration of 500 nM that was used through this research (0.027 mg/mL polymer), and the in vivo effective siRNA dose of 8 mg/kg (0.367 mg/mL polymer) [27]. Both Short and Long PGAamine:siRNA polyplexes were not toxic to RBCs at low concentrations, similarly to dextran. As it is established that high zeta potential correlates with toxicity and the recruitment of immune cells [23], Short PGAamine:siRNA polyplex was slightly more toxic than Long PGAamine:siRNA polyplex when administered at 41 μM concentration (~5% compared with ~1% hemolysis, respectively). SDS served as the positive control and caused ~14% hemolysis.

## 4. Discussion

PGAamine polymers were obtained by conjugating ethylenediamine moieties to the PGA backbone. The use of only 1.1 eq. of ethylenediamine indicated an efficient and controlled conjugation process obtained by the conjugation reagent CDI. The decreased length of the final PGAamine polymers compared with the PGA precursors can be attributed to a backbone cleavage during the acidic deprotection of Boc. Both MALS and DLS analyses indicated that two synthetic batches bearing different *M*_w_ were obtained. In accordance, the physico-chemical characteristics of the complexes obtained by mixing each batch with siRNA differed: polyplexes of Short PGAamine demonstrated higher zeta potential than polyplexes of Long PGAamine and siRNA. To elucidate the binding affinities of the two polymers with siRNA, ITC was performed. Altogether, the data obtained from ITC measurements did not reveal significant differences in the binding affinities of the two polymers with siRNA. Nevertheless, only the Long PGAamine:siRNA polyplex demonstrated silencing activity in HeLa cells, while polyplexes of Short PGAamine and siRNA failed to show specific mRNA knockdown. This length-dependent activity of PGAamine as siRNA nanocarrier was further validated in 6 other batches. Moreover, when Long PGAamine was separated to fractions, only the longer fraction demonstrated silencing activity. To evaluate whether the different levels of silencing activities are attributed to distinct internalization mechanisms that govern the internalization of Long and Short PGAamine polyplexes into HeLa cells, evaluation of the polyplexes’ colocalization with endo-lysosomal markers was performed. Confocal images indicated that both polyplexes internalized into HeLa cells and had similar ratios of colocalization with endosomes and lysosomes. We further investigated the structural characteristics of the polyplexes using MD simulation. The final structures of the polyplexes showed that Short PGAamine polymers fully covered the siRNA, while Long PGAamine polymers left part of the siRNA exposed to the surroundings. This structure of Long PGAamine:siRNA polyplex resulted in lower zeta potential, higher biological stability, and pronounced biological availability to induce gene silencing compared with polyplexes of Short PGAamine and siRNA. We, therefore, postulate that the reduced biological stability of Short PGAamine:siRNA compared with Long PGAamine:siRNA polyplexes is the dominant factor affecting the silencing activity, rather than the thermodynamic affinity as measured by ITC.

## 5. Conclusions

Polymer therapeutics can address many of the challenges raised by the delivery of RNAi to tumors, possessing great potential in the area of personalized medicine. Its clinical use, however, is hampered by the inherent heterogeneity of these therapeutic modalities. In this manuscript, we demonstrated that while Long PGAamine complexed with siRNA forms active polyplexes, complexes of Short PGAamine and siRNA are not well-suited to induce gene silencing. Even though ITC measurements demonstrated a non-significant difference in the binding affinities between the two polymer types and siRNA, the biological stabilities of these two complexes differed. MD simulation revealed that complexes of Short PGAamine and siRNA possess a structure exposing a higher amount of amine moieties to the surroundings compared to Long PGAamine:siRNA polyplexes, resulting in a higher zeta potential, leading to faster biological degradation and diminished gene silencing activity. Altogether, we demonstrated that the length of the polymer was a critical factor in determining the biological activity. Therefore, our study highlights the importance of an adequate physico-chemical characterization for the design of a tailor-made delivery system for siRNA, implying that multidisciplinary techniques should be routinely integrated in this discipline.

## Supplementary Materials

Supplementary Materials are available online at http://www.mdpi.com/2073-4360/10/5/548/s1.
