# Supplementary material for: Molecular Weight-Dependent Activity of Aminated Poly(α)glutamates as siRNA Nanocarriers
Source: Polymers (Basel). 2018 May 20;10(5):548. doi: 10.3390/polym10050548 (PMC6415365; doi:10.3390/polym10050548)
Supplement: Supplementary file 1 [file polymers-10-00548-s001.zip › polymers-302641-supplementary.pdf]

Molecular Weight-Dependent Activity of Aminated Poly(α)glutamates as siRNA Nanocarriers

Adva Krivitsky [1], Vadim Krivitsky [2], Dina Polyak [1,3], Anna Scomparin [1], Shay Eliyahu [1], Hadas Gibori [1], Eilam Yeini [1], Evgeni Pisarevsky [1], Rachel Blau [1] and Ronit Satchi-Fainaro [1,*]

1 Department of Physiology and Pharmacology, Sackler Faculty of Medicine, Room 607, Tel Aviv University, Tel Aviv 69978, Israel; advashy@gmail.com (A.K.); dina.polyak@gmail.com (D.P.); anna.scomparin@gmail.com (A.S.); s_eliahu@hotmail.com (S.E.); hadas.gibori@gmail.com (H.G.); eilamyeini@mail.tau.ac.il (E.Y.); Jalchemic@gmail.com (E.P.); rachelniss@gmail.com (R.B.)
2 School of Chemistry, the Raymond and Beverly Sackler Faculty of Exact Sciences, Tel-Aviv University, Tel Aviv 69978, Israel; vadimkri@gmail.com (V.K.).
3 Department of Neurosurgery, Stanford University School of Medicine, Stanford, CA 94305, USA (D.P.)
* Correspondence: ronitsf@post.tau.ac.il; Tel.: +972-3-640-7427

A

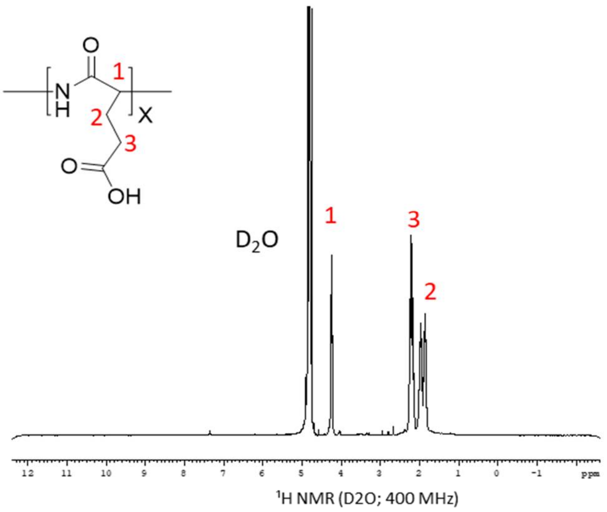

B

Polymer	Mn [g/mol]	PDI	DP
Short	11820	1.012	92
Long	21020	1.410	163

Figure S1: Characterization of the PGA precursor. (**A**) ^{1}H-NMR spectrum obtained at 400 MHz. (**B**) a table summarizing the Molecular weight (M_n), polydispersity index (PDI), and calculated degree of polymerization (DP), as obtained by MALS.

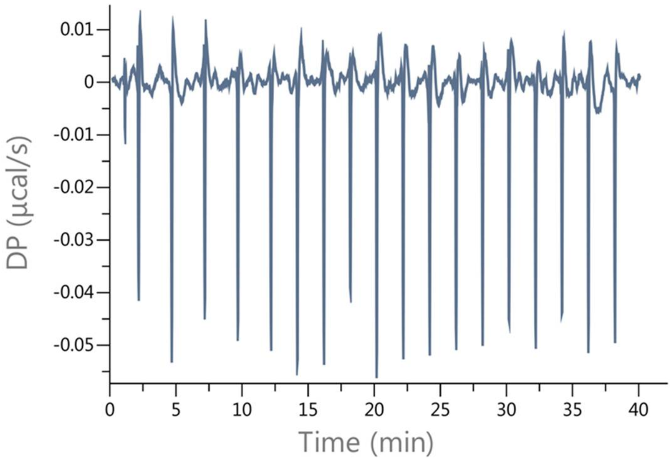

Figure S2: Isothermal titration calorimetry (ITC) of siRNA titrated into buffer. The row data obtained from a titration of 20 µM siRNA solution (760 µM Phosphate groups) into 15 mM HEPES.

A.

PGAamine batch	Mn [g/mol]	PDI	DP	Silencing activity
1	6476	1.358	23	Not active
2	7784	1.376	27	
Short	7762	1.236	27	
3	9210	1.328	32	
4	9847	1.465	35	
5	11060	1.274	39	
Long	15880	1.236	56	Active
6	20200	1.089	71	

B.

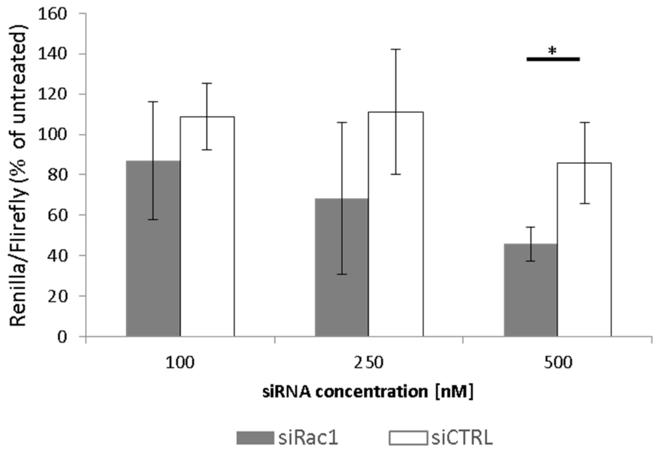

Figure S3: Various PGAamine synthetic batches. (**A**) a table summarizing the molecular weight (M_n) and polydispersity index (PDI) of various PGAamine synthetic batches, as analyzed by MALS, their calculated degree of polymerization (DP), versus the silencing activity, as tested by psiCHECK reporter assay. (**B**) The silencing activity of PGAamine batch #6, as tested by psiCHECK reporter assay. Statistical significance was determined using *t*-test, * $p < 0.05$

A.

Fraction of "Long" polymer	Mn [g/mol]	PDI	DP
Shorter	12180	1.082	43
Longer	15440	1.021	54

B.

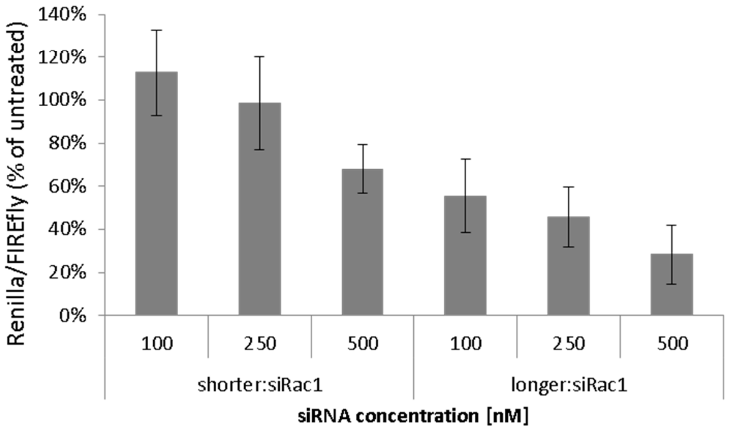

Figure S4: Separation of Long PGAamine into shorter and longer fractions. (**A**) The molecular weight (M_n), polydispersity index (PDI), and calculated degree of polymerization (DP) of the two separate fractions of "Long" PGAamine, as analyzed by MALS. (**B**) Silencing activity of the two separate fractions, as obtained by psiCHECK reporter assay.

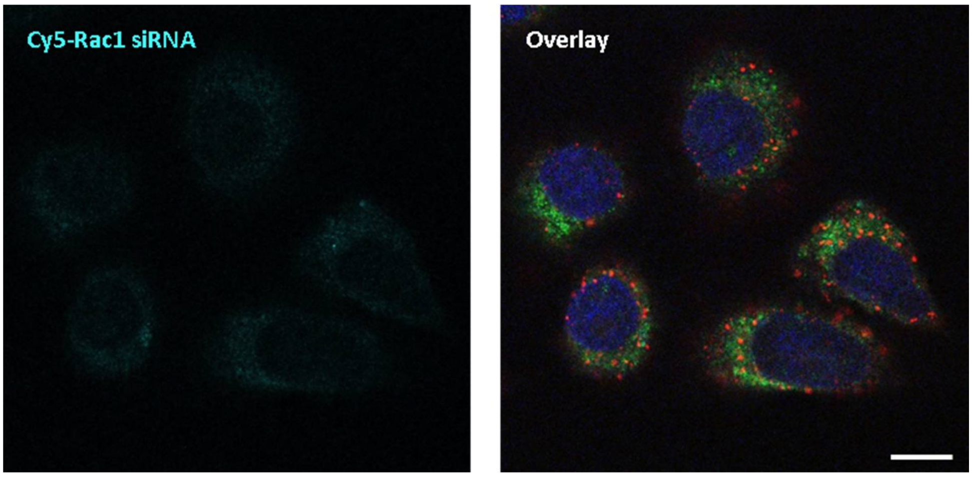

Figure S5: Cy5-Rac1 siRNA hardly internalized into HeLa cells following 24 h of treatment. HeLa cells were incubated with Cy5-Rac1 siRNA at a concentration of 100 nM. Scale bar = 10 µm. Blue-DAPI (nuclei), Red-EEA1 (endosomes), Green- LAMP1 (lysosomes), Cyan- Rac1-Cy5 siRNA.
